# Application of surgical pleth index in the opioid-free anesthesia: A randomized controlled trial

**DOI:** 10.1097/MD.0000000000035172

**Published:** 2023-10-27

**Authors:** Jingwei Dai, Duozhi Wu, Xiaoguang Cui, Shanliang Li, Fengmei Xu

**Affiliations:** a Department of Anesthesiology, Hainan Wanning People’s Hospital, Wanning, Hainan, China; b Department of Anesthesiology, Hainan General Hospital, Haikou, Hainan, China; c Department of Anesthesiology, The First Affiliated Hospital of Hainan Medical University, Haikou, Hainan, China.

**Keywords:** entropy, lower abdominal or pelvic surgery, opioid-free anesthesia, quadratus lumborum block, surgical plethy index

## Abstract

**Background:**

Currently, there is no gold standard for monitoring noxious stimulation during surgery, and the surgical pleth index (SPI) is only one of many monitoring methods. It is commonly used in the monitoring of conventional opiate anesthesia, but its effectiveness in opioid-free anesthesia (OFA) has not been evaluated. Therefore, the aim of this study was to observe the guidance value of the surgical pleth index in opioid-free anesthesia for patients undergoing lower abdominal or pelvic surgery.

**Methods:**

A total of 122 patients who underwent lower abdominal or pelvic surgery in our hospital between March 2021 and July 2022 were selected and equally divided into OFA (F) and control (C) groups according to the random number table method. Both groups underwent ultrasound-guided unilateral/bilateral quadratus lumborum block in the supine position according to the surgical field. In group F, 0.50% lidocaine and 0.20% ropivacaine (in 20 mL of 0.9% normal saline) were injected on each side. In group C, 20 mL 0.9% normal saline was injected on each side. Group F received general anesthesia without opioids and group C received general anesthesia with opioids. BP, pulse oxygen saturation, P_ET_CO_2_, reactionentropy, stateentropy, and SPI values; Steward score; dosage of propofol, dexmedetomidine, rocuronium, and diltiazem; extubation time; and awake time were monitored in both groups.

**Results:**

There were no significant differences in the general data between the 2 groups (*P* > .05). There were no significant differences in SPI values at T0, T1, T2, T3, T4, and T5 or the number of cases requiring additional remifentanil, propofol, and diltiazem between the 2 groups (*P* > .05). The stateentropy, reactionentropy, and Steward scores were higher in group F than in group C at T4 and T5, while the extubation and awake times were lower in group F than in group C (*P* < .05). The heart rate and SPI of group F were lower than that of group C at T3 (*P* < .05).

**Conclusion:**

The guiding value of SPI in OFA was similar to its use in opiated anesthesia. Its clinical efficacy is exact, vital signs are stable, enabling rapid, and complete regaining of consciousness.

## 1. Introduction

During anesthesia and surgery, noxious stimulation can activate the sympathetic neural pathways mediated by subcortical structures, thereby causing a series of changes in neurohumoral function and activating stress responses.^[1,2]^ The surgical pleth index (SPI), based on noninvasive finger pulse oxygen monitoring using a GE monitor, has recently emerged as an indicator of noxious stimulation. It integrates the normalized heartbeat interval and the photoplethysmography pulse-wave amplitude into 2 continuous cardiovascular variables. The activity of peripheral sympathetic nerves was quantified to effectively evaluate the magnitude of noxious stimulation.^[3,4]^ There are 2 pillars of opioid-free anesthesia (OFA): regional anesthesia and multimodal analgesia.^[5,6]^ Quadratus lumborum block (QLB) can reduce the dosage of sufentanil in postoperative analgesia during cesarean section, elderly hip surgery, and laparoscopic radical nephrectomy to reduce postoperative inflammation, nausea, and vomiting.^[7,8]^ SPI-guided analgesia can improve the quality of recovery 24 hours after femoral internal fixation in elderly patients.^[9]^ During the induction of general anesthesia in patients undergoing cardiac valve replacement, SPI was used as the target to adjust the dosage of analgesic drugs to stabilize hemodynamics during the induction process, improve safety, and guide rational clinical drug use.^[10]^ However, there is no gold standard for monitoring nociception, and it is unclear whether the SPI can be used to assess nociception during OFA. The purpose of this study was to evaluate the value of SPI for guiding OFA, in order to provide some reference for the monitoring and evaluation of noxious stimulation in OFA.

## 2. Materials and methods

### 2.1. Ethics approval and patient selection

This was a prospective study and was approved by the Institutional Ethics Committee of Hainan Wanning People’s Hospital (SL-2021-002). The inclusion criteria were as follows: (1) American Society of Anesthesiologists Class I to III, aged ≥ 18 years; (2) normal liver and kidney function; (3) undergoing open or laparoscopic lower abdominal or pelvic surgery; and (4) no history of allergy to the drugs used in this study. Patients or their families provided written informed consent before the trial and could withdraw from the trial at any stage. All methods were performed in accordance with the relevant guidelines, regulations, and CONSORT recommendations. This trial was registered with www.medicalresearch.org.cn (reg. No. MR-46-23-010135) and https://www.chictr.org.cn (reg. No. ChiCTR2300072842). Supplementary registration.

### 2.2. Exclusion criteria

The exclusion criteria were as follows: (1) did not sign the informed consent form, (2) severe circulatory dysfunction or arrhythmia, especially bradyarrhythmia, (3) use of sedative or antipsychotic drugs within 30 days before surgery,^[11]^ (4) pacemaker implantation or long-term oral administration of β-blockers before surgery.

### 2.3. Randomization, blinding, and data collection

**Figure 1.**
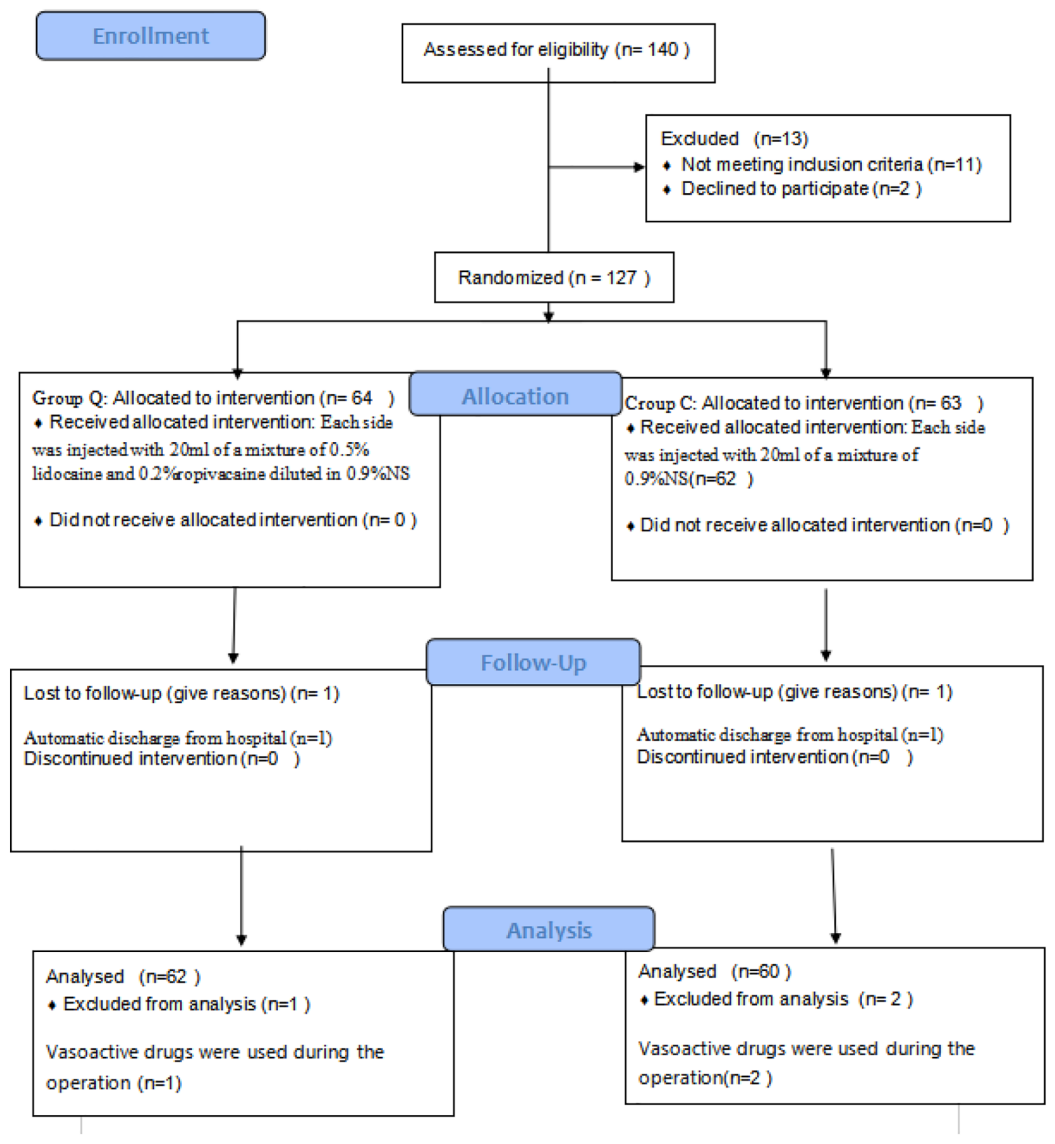
Consort e-flowchart.

All patients were divided into 2 groups by the same anesthesia nurse according to the random number table method. The remainder was obtained by dividing the random number in the random number table by the number of groups, while the aliquot was the control group and the experimental group with the remainder, with 40 mL of unlabeled local anesthetic or 0.9% NS in a marked 1 sealed envelope and the group label in a marked 2 sealed envelope, handed to the anesthesiologist on duty, and the patients were blinded to the group assignment. Two patients refused to participate, 11 patients were excluded according to the exclusion criteria, 2 patients were lost to follow up, and 3 patients used vasoactive agents during the procedure, resulting in 122 patients being enrolled: 62 in the OFA (F) group and 60 in the control (C) group (Fig.1).

### 2.4. Intervention protocols

**Figure 2.**
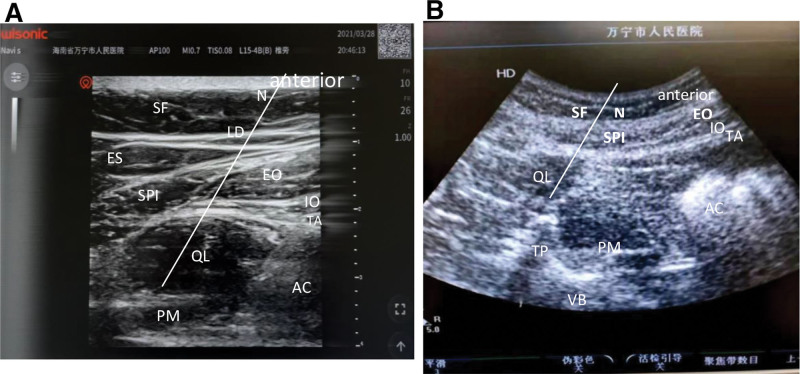
(A)“Eye sign” and (B) “baby sign”. SF = subcutaneous fat; LD = latissimus dorsi; ES = vertical ridge; SPI = serratus posteriori; EO = external oblique muscle; IO = internal oblique muscle; TA = transversus abdominis; QL = quadratus lumborum; PM = psoas major; TP = transverse process; VB = vertebral body; AC = abdominal cavity; N = needle indication; “eye sign” = SPI-eyebrow, QL—eyeball, The 3 layers of abdominal wall muscles—crow’s feet; “baby sign” = QL—infant head, PM—infant body, TP and VB—pillow and cradle.

After the patients entered the operating room, a Datex-Ohmeda anesthesia monitor was connected to monitor the entropy index. The stateentropy (SE), reactionentropy (RE), and SPI values were monitored using a GE Healthcare monitor (Helsinki, Finland). Both groups received target-controlled infusion (TCI) of dexmedetomidine (Dex) 0.8 μg/kg/10 minutes, intravenous tropisetron 5 mg, and intramuscular penehyclidine hydrochloride 0.1 mg/kg. After sedation, “eye sign” (Fig. 2A) and “infantile sign”(Fig. 2B) changes were assessed. According to the requirements of the surgical field, unilateral/bilateral QLB surgery was performed through the anterior approach under local anesthesia with ultrasound guidance. After the target site was determined, 0.50% lidocaine (Hebei Tiancheng Pharmaceutical Co., Ltd., Cangzhou, Hebei, China; approval number: H13022313; specification: 5 mL: 100 mg) and 0.20% ropivacaine (Shijiazhuang Siyao Co., Limited, Shijiazhuang, Hebei, China; approval number: H20203107; specification: 10 mL: 100 mg) in 20 ml 0.9% normal saline were injected into each side of group F. In group C, 20 mL 0.9% normal saline was injected on each side. Fifteen minutes after administration, the level of sensory block was assessed in all subjects using acupuncture.^[12]^

After testing the block level, the anesthesiologist on duty opened the sealed envelope marked 2 and decided whether to administer the OFA based on the block plane test results. Total intravenous anesthesia was used in both groups. Anesthesia was induced and maintained with TCI of propofol 3 to 3.5 μg/mL and intravenous rocuronium 0.6 mg/kg in group F, or with TCI of propofol 3 to 3.5 μg/mL and remifentanil (Yichang Humanwell Pharmaceutical Co. Ltd., Yichang, Hubei, China; approval number: H20030197; specification:1 mg) 2 to 4 ng/mL and intravenous rocuronium 0.6 mg/kg in group C. Then, the 2 groups were fitted with a laryngeal mask airway 3 to 5 according to body weight, and 0.3 mg/kg rocuronium was added intermittently according to the need of surgery to maintain the entropy index value of 40 to 65 and SPI value of 30 to 50.

When blood pressure was low and the heart rate was slow, ephedrine and atropine were administered for symptomatic treatment. Patients in both groups received intravenous flurbiprofen axetil 50 mg during skin suturing, followed by patient-controlled intravenous analgesia with esketamine 0.015 mg/(kg·h) (total dose ≤ 50 mg) + flurbiprofen 200 mg + Dex 100μg + tropisetron 5 mg + 0.9%NS to 100 mL, 2 mL/h. Electrocardiography, BP, pulse oxygen saturation (SPO_2_), P_ET_CO_2_, SE, RE, SPI, Steward score, extubation time, awake time, as well as the propofol, Dex, rocuronium, and diltiazem dosages were monitored in both groups.

### 2.5. Determination of the anesthetic effect

1. The patient’s tolerance to the anesthesia regimen and allergic reaction were evaluated by comparing the hemodynamic status.^[13]^ Blood pressure > 140/90 mm Hg and heart rate > 140 bpm for more than 30 s were considered too shallow for anesthesia.
2. When the values of SE and RE were both >65 or the difference was >10, it reflected the recovery of pain or muscle relaxation, and anesthesia was considered insufficient.
3. An amplitude of SPI change >10 or SPI value >50 was considered to indicate insufficient analgesia.

When one or more of these conditions were met, propofol (1 mg/kg) was rapidly pumped and the patient was observed for 5 minutes. If it was ineffective, diltiazem (Beijing SHKB Pharmaceutical Co. Ltd., Beijing, China; approval number: approved Chinese medicine H20031228; specification:10 mg) 0.2 mg/kg total dose ≤ 10 mg was given intravenously and the patient was observed for 5 minutes. The failure of OFA was defined as ineffective sequential use of the 2 treatments, and remifentanil 2 to 4 ng/mL was injected timely as a rescue.

### 2.6. Study endpoints and measurements

The primary endpoints of this study were the values of SPI, SE, RE, systolic blood pressure (SBP), diastolic blood pressure (DBP), SPO2 and Steward score before induction (T0), after induction (T1), at skin incision (T2), 1 hour during operation (T3), at extubation (T4), and after leaving the room (T5) in the 2 groups. The secondary endpoints included the dosages of propofol, rocuronium, and Dex, anesthesia time, extubation time, awake time, as well as the number of patients who needed additional propofol, remifentanil, and diltiazem during surgery in the 2 groups.

### 2.7. Sample size calculation

This study was prospective, and the sample size was calculated using the formula:


$$\begin{array}{c} n=\frac{{\left(Z_{\alpha }+Z_{\beta }\right)}^{2}(1+1/k)\;p(1-p)}{{\left(P_{e}-P_{c}\right)}^{2}},p=\frac{p_{e}+p_{c}}{1+k} \end{array}$$

where *α* = 0.05, *β* = 0.20, *k* is the ratio of the control to experimental group, *k* = 1. *p*_*e*_ = 0.87 is the measured probability value of the preexperiment, and *P*_*c*_ = 1.00 is the measured probability value of the control group. The Z_α_ and Z_β_-score can be found in the Z-score table. The sample size of the trial was appropriately increased by 10% to 20% to account for the influence of factors such as exclusion and loss to follow-up. This study included 140 patients who underwent lower abdominal or pelvic surgery at our hospital between March 2021 and July 2022.

### 2.8. Statistical analysis

SPSS 25.0 statistical software (IBM Corp., Armonk, NY) was used for data analysis, and the measured data were expressed as means ± standard deviations (*x̄*±*s*). Student *t*-test was used to compare differences in continuous variables between the 2 groups. Repeated analysis of variance was used for repeated measurements, and statistical data were expressed as frequencies and compared between groups using Pearson *χ*^2^ test. Differences with *P* < .05 were considered statistically significant.

## 3. Results

### 3.1. Comparison of general data between the 2 groups

There were no significant differences in sex, age, BMI, surgical method, department, preoperative complications, or block point between the 2 groups (*P* > .05 in all cases; Table 1).

**Table 1:** Comparison of general data between the 2 groups (*x̄*±*s*).

Project/group	Gender (cases)	Age	BMI	Type of operation (cases)	Department (cases)			Before surgery Comorbidity(cases) Yes/No	Point of arrest Unilateral/bilateral (cases)
	(Male/female)	(years)	(kg/m·m)	(Laparoscopic/open)	Gynecology	General surgery	Urology		
F group (n = 62)	30/32	51.50 ± 16.79	23.27 ± 3.62	40/22	16	20	26	41/21	19/43
C group (n = 60)	36/24	54.23 ± 13.33	22.81 ± 3.87	41/19	15	17	28	36/24	15/45
* t/χ^2^*	1.656	−0.993	0.678	0.199	0.010	0.222	0.277	0.492	0.483
* P*	0.198	0.323	0.499	0.655	0.919	0.637	0.599	0.483	0.487

### 3.2. Comparison of BP, heart rate (HR), SPO_2_, SE, RE, SPI, and Steward score before and after treatment between the 2 groups

There were no significant differences in SBP, DBP, HR, SPO_2_, SE, RE, SPI, or Steward score between the 2 groups at T0 (*P* > .05). There were no significant differences in SPI and SPO_2_ between the 2 groups at T0, T1, T2, T3, T4, or T5 (*P* > .05). The SBP and DBP values were significantly higher in group F than in group C at T1 (*P* = .003; *P* = .021, respectively). The HR and SPI of group F were lower than that of group C at T3 (*P* = .017; *P* = .031, respectively). The SE and RE values as well as the Steward scores of group F were significantly higher than those of group C at T4 and T5 (*P* = .021, *P* = .001, *P* = .014; *P* = .001, *P* = .000, *P* = .032, respectively). For intragroup comparison, there were no significant difference in the values of SPI between T0 and T5 in group F or SPI between T0 and T5 in group C (*P* = .098; *P* = .876, respectively) (Table 2).

**Table 2:** Comparison of blood pressure, heart rate, SPO_2_ (%), RE, SE, SPI values, and Steward score between the 2 groups(*x̄*±*s*).

Project/group	Time	C group (n = 60)	F group (n = 62)	*t*	*P*
SBP (mm Hg)	T0	130.97 ± 18.22	133.5 ± 16.31	−0.810	.420
	T1	111.77 ± 15.10	120.23 ± 16.00	−3.001	.003
	T2	117.42 ± 17.29	119.97 ± 16.43	−0.836	.405
	T3	112.25 ± 13.44	114.69 ± 11.38	−1.085	.280
	T4	126.83 ± 13.96	127.02 ± 15.01	−0.070	.945
	T5	118.73 ± 12.23	120.07 ± 14.41	−0.553	.581
DBP (mm Hg)	T0	78.10 ± 9.29	79.26 ± 9.81	−0.669	.505
	T1	67.82 ± 11.00	72.70 ± 12.00	−2.346	.021
	T2	71.52 ± 10.67	74.37 ± 11.50	−1.420	.158
	T3	70.68 ± 8.88	71.66 ± 9.67	−0.581	.562
	T4	75.82 ± 11.91	77.39 ± 11.15	−0.752	.453
	T5	70.82 ± 12.07	72.90 ± 12.13	−0.949	.344
HR (bpm)	T0	86.02 ± 17.14	81.23 ± 12.22	1.773	.079
	T1	79.15 ± 16.17	77.50 ± 12.72	0.628	.531
	T2	75.53 ± 14.46	73.35 ± 10.93	0.941	.349
	T3	74.22 ± 13.66	68.90 ± 10.37	2.424	.017
	T4	83.65 ± 14.23	80.00 ± 12.35	1.515	.132
	T5	72.43 ± 12.06	70.55 ± 10.91	0.904	.368
SPO2 (%)	T0	97.88 ± 1.90	97.84 ± 2.51	0.111	.912
	T1	99.97 ± 0.18	99.85 ± 0.47	1.732	.087
	T2	99.98 ± 0.13	99.95 ± 0.38	0.612	.542
	T3	100.00 ± 0.00	100.00 ± 0.00	/	/
	T4	100.00 ± 0.00	99.85 ± 0.67	1.696	.095
	T5	98.05 ± 1.78	98.20 ± 0.98	−0.579	.564
SPI	T0	76.97 ± 8.86	79.61 ± 8.85	−1.843	.068
	T1	31.47 ± 5.65	32.98 ± 6.00	−1.437	.153
	T2	47.95 ± 7.98	47.58 ± 5.76	0.294	.769
	T3	37.07 ± 4.41	35.35 ± 4.27	2.179	.031
	T4	58.81 ± 6.80	60.85 ± 6.76	−1.661	.099
	T5	74.03 ± 9.39	75.44 ± 7.46	−0.915	.362
SE	T0	85.00 ± 2.97	85.50 ± 2.63	−0.985	.327
	T1	41.12 ± 2.74	41.77 ± 3.29	−1.198	.233
	T2	42.43 ± 2.51	42.13 ± 2.97	0.610	.543
	T3	41.47 ± 1.35	41.50 ± 1.80	−0.116	.908
	T4	83.52 ± 1.96	84.44 ± 2.35	−2.340	.021
	T5	83.71 ± 1.93	84.91 ± 2.57	−3.370	.001
RE	T0	94.23 ± 2.12	94.55 ± 2.14	−0.817	.416
	T1	43.38 ± 4.15	43.89 ± 4.68	−0.628	.531
	T2	48.18 ± 7.05	47.05 ± 6.43	0.929	.355
	T3	42.48 ± 1.96	42.26 ± 2.06	0.618	.538
	T4	92.03 ± 1.83	93.24 ± 1.99	−3.488	.001
	T5	93.73 ± 1.92	95.31 ± 1.58	−4.970	.000
Steward	T0	5.11 ± 0.87	5.04 ± 0.95	0.424	.672
	T1	/	/	/	/
	T2	/	/	/	/
	T3	/	/	/	/
	T4	4.03 ± 0.95	4.25 ± 1.17	−2.483	.014
	T5	4.37 ± 0.77	4.74 ± 1.08	−2.173	.032

### 3.3. Comparison of intraoperative symptomatic treatment between the 2 groups

There was no significant difference in the number of patients who required remifentanil, propofol, or diltiazem add on treatment during surgery between the 2 groups (*P* > .05) (Table 3).

**Table 3:** The number of cases requiring addition of remifentanil, propofol, and diltiazem during the operation (%,case).

Project/group	Remifentanil	Propofol	Diltiazem
Fgroup (n = 62)	1.612 (1)	0.113 (7)	0.048 (3)
Cgroup (n = 60)	0.000 (0)	0.083 (5)	0.033 (2)
* X^2^*	0.976	0.300	0.176
* P*	0.323	0.583	0.675

### 3.4. Comparison of the consumption of propofol, rocuronium, and Dex, anesthesia time, extubation time, and awake time between the 2 groups

There were no significant differences in the dosage of propofol, rocuronium, and Dex, or anesthesia duration between the 2 groups (*P *> .05). The extubation and awake times were significantly shorter in group F than in group C (*P* = .008; *P* = .007 respectively) (Table 4).

**Table 4:** Comparison of dosage of propofol, rocuronium, dexmedetomidine, duration of anesthesia, extubation time and awake time between the 2 groups (*x̄*±*s*).

Project/group	Propofol (mg)	Rocuronium (mg)	Dex (ug)	Duration of anesthesia (min)	Extubation time (min)	Awake time(min)
Fgroup (n = 62)	797.34 ± 472.76	73.44 ± 33.58	37.42 ± 18.21	135.23 ± 84.98	8.86 ± 4.89	22.54 ± 10.34
Cgroup (n = 60)	675.00 ± 377.46	67.42 ± 26.48	32.55 ± 11.04	112.45 ± 72.83	18.00 ± 18.65	34.63 ± 23.34
* t*	−1.576	−1.097	−1.094	−1.587	2.808	2.814
* P*	0.115	0.275	0.076	0.115	0.008	0.007

## 4. Discussion

Nociception is defined as the neural encoding of actual or imminent tissue damage (such as noxious stimuli), whereas pain is a subjective experience associated with imminent or actual injury.^[14]^ Unconscious patients do not recall pain, and increasing data suggests that surgical stress, sympathetic nerve activation, inflammatory responses, and the immunosuppressive effects of anesthesia can be managed by treatments other than opioids.^[15,16]^ Animal experiments have shown that QLB is an effective and safe alternative to opioids for providing adequate analgesia during and after ovariectomy in dogs,^[17]^ as it can inhibit somatic and visceral pain.^[18]^ Hakim et al^[19]^ found that in gynecological laparoscopic surgery, the QoR-40 scale score of the group that did not receive opioids was approximately 10 points higher than that of the group that received opioids 24 hours after surgery. Moreover, the difference was statistically significant (*P* < .05), suggesting that the use of opioids during surgery can reduce the quality of postoperative recovery. However, owing to the lack of clinical indicators for intraoperative analgesia, the use of analgesic drugs is often inferred based on the patient’s body movements, blood pressure, and heart rate, which have poor specificity and can easily result in excessive or insufficient administration of analgesic drugs. SPI, which is calculated from the pulse photoplethysmography amplitude and heart rate interval derived from SPO_2_ measurements, has been used clinically to monitor the balance between nociceptive and antinociceptive stimuli during general anesthesia.^[20–22]^ A study of craniocerebral surgery using general anesthesia combined with bilateral scalp block showed that the SPI appeared to be more reliable than the analgesia nociception index and that the SPI was not affected by bleeding or norepinephrine infusion.^[23]^

The patients’ SE and RE were used to evaluate the depth of anesthesia, and SPI was used to evaluate the central nervous system response to pain “nociception” during surgery.^[24–26]^ Studies have shown that type II pectoral nerve block for breast surgery^[27]^ and abdominal wall block (including rectus sheath block and QLB) for single-port laparoscopic hernia surgery^[28]^ under the guidance of SPI can reduce the consumption of remifentanil and pain scores during general anesthesia. SPI can be a cost effective and reliable option during general anesthesia.^[29]^ In this study, we found that the anesthesia time as well as the dosage of propofol, rocuronium, and Dex were not statistically different, while SPI, SE, RE, SBP, and DBP changed with the depth of anesthesia and the intensity of surgical stimulation. However, there was no statistically significant difference between the 2 groups. There was no significant difference in SPI values between the 2 groups at T0, T1, T2, T3, T4, and T5, indicating that SPI predicted the effect of OFA and opioid anesthesia on nociception with similar efficacy. In a prospective study of 26 patients undergoing rotator cuff repair under general anesthesia, Wennervirta et al found that SPI was significantly lower in the group undergoing general anesthesia combined with interscalene nerve block than in the group undergoing general anesthesia alone, which confirmed that SPI was superior to heart rate, blood pressure, and reactive entropy as an intraoperative measure of pain-analgesia balance.^[30]^ SPI can be used to monitor noxious stimuli during general anesthesia combined with local anesthesia, nerve block and multimodal analgesia during surgery. SPI-guided multimodal analgesia during laparoscopic cholecystectomy under total intravenous anesthesia [0.75% ropivacaine (3 mL) was used for local infiltration anesthesia in each endoscopic incision before surgery; Intravenous parecoxib sodium (40 mg) was given 1 minute after tracheal intubation for preemptive analgesia], which required a lower dose of fentanyl and resulted in a shorter extubation time.^[31]^ In addition, there was no difference in SPI between the 2 groups at T1, which may be due to reduced stimulation during laryngeal mask airway compared to endotracheal tube insertion.

The SE is calculated based on an electroencephalogram, offering a comprehensive index of electroencephalogram and frontal muscle electromyography that can better reflect the depth of anesthesia. The entropy index and cerebral oxygen metabolism are independently associated with postoperative hyperalgesia.^[32]^ In this trial, the SE and RE values as well as the Steward scores of group F were higher than those of group C at T4 and T5, indicating that group F avoided the sedative effect of opioids, became fully awake, and spent less time under the effects of anesthesia. In this experiment, the SBP and DBP values of group F were higher than those of group C at T1, indicating that anesthesia induction in group F became more stable by avoiding the inhibition of sympathetic nerves by opioids. The HR and SPI of group F were lower than that of group C at T3, indicating that the full onset time of QLB was slow, requiring 20 to 30 minutes.^[33]^ This suggests that QLB provides visceral and somatic analgesia for lower abdominal or pelvic surgery comparable to the analgesic effects of opioids. This somatic and visceral analgesia effect of peripheral nerve block may be superior to the central analgesia of opioids.

The limitations of this study are as follows: 1. the effect of position on the SPI was not excluded. 2. This was not a multicenter study. 3. Further studies are needed to evaluate the efficacy of SPI for monitoring noxious stimuli during OFA.

In conclusion, SPI is as effective as in guiding OFA as in opiate anesthesia, with definite clinical efficacy and stable anesthesia induction. The SE, RE, and Steward scores were higher at extubation and discharge, while the extubation and awake times were shorter.

## Acknowledgments

This study was supported by the Department of Anesthesiology of the Hainan Wanning People’s Hospital, Wanning, Hainan, China.

## Author contributions

**Conceptualization:** Duozhi Wu.

**Investigation:** Fengmei Xu.

**Software:** Shanliang Li.

**Supervision:** Xiaoguang Cui.

**Writing – original draft:** Jingwei Dai.

**Writing – review & editing:** Jingwei Dai, Duozhi Wu.
